# Process-Resolved VOC Source Profiles from Typical Industries in Deyang and Their Implications for Regional Composite Profiles in the Chengdu–Chongqing Region

**DOI:** 10.3390/toxics14050423

**Published:** 2026-05-12

**Authors:** Xiao Hu, Yuxuan Huang, Xiaohan Shao, Yuehua Liu, Tingting Peng, Bo Zhu, Jianzhang Huang, Hanyang Man

**Affiliations:** 1 Fujian Key Laboratory of Pollution Control & Resource Reuse, College of Environmental and Resource Sciences, Fujian Normal University, Fuzhou 350007, China; 2 Guangdong Suncere Technology Co., Ltd., Guangzhou 510700, China

**Keywords:** volatile organic compounds, process-resolved source profiles, industrial emissions, OH reactivity, regional composite profile

## Abstract

Volatile organic compound (VOC) emissions exhibit strong process-level heterogeneity, yet regional source characterization still commonly relies on sector-average profiles, introducing substantial uncertainty into source identification and control prioritization. In this study, process-resolved VOC source profiles were established for five representative industrial sectors in Deyang, a typical industrial city in the Chengdu–Chongqing region, including pharmaceutical manufacturing, industrial coating, chemical industry, food manufacturing, and the textile industry. A total of 19 organized emission samples were collected from 9 enterprises, and 123 VOC species were quantified. These measured profiles were further integrated with literature-derived profiles and a bottom-up emission inventory to construct an emission-weighted regional composite source profile for 17 major industrial sectors. An emission-based hydroxyl radical (OH) reactivity-weighted framework was then introduced to compare mass-dominant and chemically dominant VOC sources. The results showed pronounced process- and sector-specific differences in composition. Pharmaceutical manufacturing was mainly dominated by oxygenated VOCs (OVOCs), industrial coating by low-carbon halocarbons, the chemical industry by methanol and reactive low-carbon compounds, food manufacturing by alkenes and OVOCs, and the textile industry by light alkanes. At the regional scale, industrial VOC emissions were dominated by OVOCs (35.67%), followed by alkanes (19.01%) and aromatics (15.99%). Ethyl acetate, 1,4-dioxane, 1,1,2,2-tetrachloroethane, and m/p-xylene were identified as the most abundant species. However, OH reactivity was largely dominated by alkenes, and substantial discrepancies were observed between emission contribution and OH-reactivity-weighted contribution across sectors. In particular, the chemical industry contributed 21.10 ± 8.43% of reactive organic gas emissions but 28.82 ± 11.61% of OH-weighted emissions, whereas printing contributed 13.55 ± 13.42% of mass emissions but only 7.66 ± 13.08% of OH-weighted emissions. These findings demonstrate that regional VOC management should move beyond bulk mass reduction and prioritize high-reactivity sectors and process units to maximize O_3_ mitigation benefits.

## 1. Introduction

Anthropogenic volatile organic compounds (VOCs) are key precursors of ozone (O_3_) and secondary organic aerosol (SOA), making their control central to mitigating complex air pollution in urban and industrial regions [1,2,3]. However, the atmospheric impacts of VOCs depend crucially not only on total emission magnitude but also on chemical composition, as different species vary widely in O_3_ formation potential, OH reactivity, SOA production, and toxicity [4,5,6,7,8]. This underscores the importance of composition-resolved source characterization for effective VOC control.

This challenge is further amplified by the strong heterogeneity of industrial VOC emissions. Even within the same broad sector, differences in process configuration, raw-material formulation, product type, and control technology can lead to substantially different species profiles. For example, distinct VOC fingerprints have been reported among petrochemical units, including atmospheric and vacuum distillation, fluid catalytic cracking, and delayed coking [9]. In industrial coating, the relative abundance of aromatics and OVOCs varies markedly across primer and topcoat applications, as well as during spraying and baking [10]. Similar composition shifts have also been observed in the printing and packaging industry when solvent-based, water-based, and UV-curing systems are used [10,11]. These process-level differences can propagate to the regional scale, yielding distinct composite source signatures among industrial regions with contrasting manufacturing structures.

Regional VOC composition commonly relies on sector-average or literature-averaged profiles, which assume sector emissions remain homogeneous and stable over time. This simplification introduces substantial uncertainty [12]. Regional mixtures do not simply combine generic source spectra; instead, emission strength and process-specific composition across sub-sectors determine them [13]. Technological upgrades, raw material substitution, product restructuring, and evolving end-of-pipe controls further affect this coupling. Thus, even within the same sector, VOC composition and tracers often differ substantially across regions and over time [14]. Although researchers increasingly recognize this heterogeneity, few process-resolved frameworks systematically integrate process-level profiles into regional composite spectra.

The Chengdu–Chongqing region is one of China’s most pollution-sensitive urban agglomerations. Its basin topography, stagnant weather, high humidity, and strong photochemistry lead to the accumulation of pollutants and co-pollution by fine particulate matter (PM_2.5_) and O_3_ [15,16,17,18]. These conditions mean that uncertainties in industrial VOC composition can bias the identification of priority species and sectors for control. Decision-makers need a more regional characterization of VOC emissions that considers both emission mass and atmospheric reactivity. Researchers increasingly recognize this heterogeneity, yet few process-resolved frameworks have systematically integrated process-level profiles into a regional composite source profile.

In this study, we analyzed industrial VOC emissions in the Chengdu–Chongqing region, focusing on five key sectors: pharmaceutical manufacturing, industrial coating, the chemical industry, food manufacturing, and the textile industry. We collected VOC data from 19 organized emission samples and combined these with published source profiles and emission inventory details. This approach allows us to analyze how process heterogeneity and regional structure influence VOC emission characteristics. We built a hierarchical framework that integrates process-level profiles into sectoral and regional composite spectra. We used the hydroxyl radical loss rate as a reactivity metric to create an OH-reactivity-weighted source profile that reflects both emission size and atmospheric activity. By comparing emission-weighted and reactivity-weighted profiles, we highlight shifts in the ranking of key VOC species and sectors and provide a foundation for shifting VOC management from mass-based to reactivity-based control. Focused on a specific region, this study provides a novel, transferable methodology. The hierarchical framework serves as a universal tool to prioritize high-reactivity sources and guide targeted VOC control strategies.

## 2. Materials and Methods

### 2.1. Study Area and Sampling Period

The Chengdu–Chongqing region is one of the most important industrial agglomerations in western China and a region that suffers from complex air pollution, where anthropogenic VOC emissions play an important role in regional O_3_ formation. Deyang, located in the northeastern Chengdu Plain and approximately 70 km from Chengdu, is a key industrial city within the Chengdu–Chongqing economic circle. According to local statistical data, the industrial added value accounted for 52% of the gross regional product in 2022 [19]. Its industrial structure is characterized by substantial VOC-emitting activities in pharmaceutical manufacturing, industrial coating, the chemical industry, food manufacturing, and textile production and is therefore broadly representative of industrial emission characteristics in the Chengdu–Chongqing region. Although field sampling was limited to Deyang, the regional composite source profile (Section 2.4) integrates these measurements with literature profiles and a sector-wide emission inventory. Thus, the regional results are not a simple extrapolation from the sampled enterprises alone, and the methodological framework is transferable to other industrial agglomerations.

**Figure 1:**
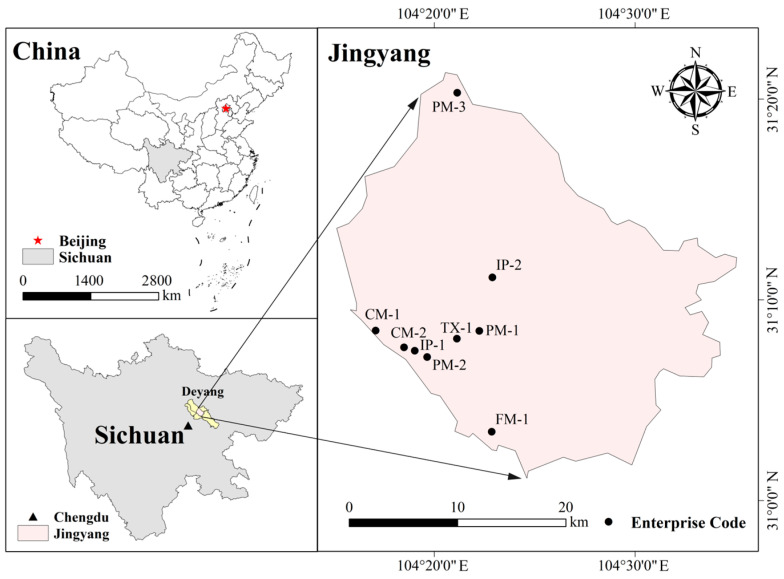
Sampling locations of investigated enterprises in Deyang, Sichuan Province.

Field sampling was conducted from 14 August to 6 September 2023 in industrial clusters located in Jingyang District, Deyang, Sichuan Province (Figure 1). This study focused primarily on organized emission sources, i.e., fixed exhaust stacks associated with major production processes. Based on the provincial list of key VOC-emitting industries in Sichuan, sampling targets were selected according to the following criteria: (1) production processes with relatively high VOC emission intensity within each industry; (2) process units representative of mainstream industrial technologies; and (3) large-scale enterprises with leading production capacity in Deyang, operating under normal production conditions and equipped with functioning end-of-pipe control facilities, such as regenerative thermal oxidizers (RTOs) or activated carbon adsorption systems. In total, 19 organized emission points were established in 9 representative enterprises, covering core VOC-emitting processes in five major industries (Table 1). The heights of the ventilation shafts varied between 10 and 15 m across different facilities.

### 2.2. Sample Collection

To characterize VOC composition from organized industrial emissions, stack gas samples were collected using both Tedlar bag sampling and synchronized Tedlar bag–SUMMA canister sampling. During the sampling campaign, meteorological conditions were generally stable, with sunny weather, ambient temperatures of 25–32 °C, a relative humidity of 44–77%, and wind speeds of 0.83–5.28 m/s, with prevailing winds from the southeast and southwest.

For stack sampling, stainless-steel tubing was used to connect the sampling system to the exhaust port. To reduce the temperature of the sampled gas and stabilize the flow rate, the tubing was coiled to extend the flow path before collection. Sampling was conducted through standard monitoring ports along the exhaust ducts while the production lines and air pollution control facilities were operating normally. Each sampling event lasted approximately 2 min. For continuous steady-state processes, one sample was collected; for batch or variable processes, duplicate samples were taken to ensure representativeness.

In addition, to assist in understanding VOC composition characteristics within typical process units, selected fugitive emission samples were collected instantaneously using SUMMA canisters (Entech Instruments Inc., Simi Valley, CA, USA), also with a sampling duration of 2 min. These supplementary samples were used only for process-level compositional reference and were not included in the construction of the organized-emission source profiles unless otherwise specified.

### 2.3. Chemical Analysis

#### 2.3.1. Analytical Procedures

Sample analysis consisted of field screening and laboratory determination. For samples collected in gas bags, total non-methane hydrocarbons (NMHCs) were measured on site using a non-methane hydrocarbon analyzer (Signal SOLAR, Signal Group Ltd., Camberley, UK). To avoid detector saturation and ensure consistency in subsequent laboratory analysis, samples with high VOC levels were diluted with high-purity nitrogen (99.999%) to approximately 1 ppm and then transferred into SUMMA canisters for storage.

Laboratory analysis of canister samples was performed according to USEPA Methods TO-14 and TO-15. VOC species were identified and quantified using a cryogenic preconcentration system (−40 °C, −60 °C, and −170 °C) coupled with gas chromatography–mass spectrometry and flame ionization detection (GC–MSD/FID, 7890B-5977E, Agilent, Santa Clara, CA, USA). A total of 123 VOC species were quantified, including 27 alkanes, 11 alkenes, 1 alkyne, 17 aromatics, 38 halocarbons, 28 oxygenated OVOCs, and 1 sulfur-containing compound (carbon disulfide). Because m-xylene and p-xylene could not be fully separated due to their similar physicochemical properties and chromatographic behavior, they were reported jointly as m/p-xylene.

#### 2.3.2. Quality Assurance and Quality Control

Strict quality assurance and quality control (QA/QC) procedures were implemented throughout sampling, storage, and analysis. Field sampling followed the Technical Specifications for Monitoring of Fugitive Emission from Fixed Sources and the Technical Specifications of Quality Assurance and Quality Control for Monitoring of Stationary Pollution Sources (Trial) [20,21]. Silanized SUMMA canisters (3.2 L) equipped with flow controllers were used for sample collection. Prior to sampling, all canisters were cleaned with high-purity nitrogen, evacuated to a residual pressure of <30 mTorr, and tested for vacuum integrity and leak tightness.

All samples were analyzed within two weeks after collection. Before laboratory analysis, the instrument was calibrated using standard gas mixtures (1 ppm, Linde Gas, Inc., Danbury, CT, USA) that covered the target VOC species. Among the 123 target compounds, 94 species showed calibration coefficients of determination (R^2^) greater than 0.99, and replicate analyses showed good consistency. Method blanks, parallel samples, and routine instrument performance checks were used to ensure data reliability throughout the analytical campaign.

### 2.4. Construction of the Regional Composite Source Profile

To characterize the overall VOC composition of industrial emissions in the Chengdu–Chongqing region, a regional composite source profile was constructed using an emission-weighted averaging approach. The source profile database included measured species profiles from the five typical industries investigated in this study, together with representative source profiles for an additional 12 industrial sectors compiled from the literature (Table 2). This study derived the weighting factors from a bottom-up emission inventory based on an enterprise-to-region hierarchy, thereby enhancing the spatial and sectoral accuracy of the emission estimates.

To avoid double-counting, the industrial coating category was defined here as coating-related process links separated from broader manufacturing sectors, and its emissions were calculated independently from those of product-manufacturing industries. The regional composite source profile was calculated as follows. This emission-weighted integration approach combines measured source profiles, literature-based profiles, and sectoral VOC emission weights to obtain the regional composite source profile.

(1) $${\;P}_{i}=\frac{E_{j}\times F_{ij}}{E_{j}}$$

where $P_{i}$ represents the mass fraction of species *i* in the regional composite source profile, %; $E_{j}$ is the emissions of industry *j*, tons/year; and $F_{ij}$ is the mass fraction of species *i* in the source profile of industry *j*, %.

### 2.5. Calculation of OH Reactivity-Weighted Emission Characteristics

To evaluate the atmospheric environmental impact of industrial VOC, we used OH reactivity (OHR) to characterize the chemical activity of the emission mixtures (with units of s^−1^). In this study, rather than calculating ambient OHR directly from observed atmospheric concentrations, we derived an emission-based reactivity metric to quantify the relative contribution of different VOC species and industrial sectors to potential OH consumption.

(2) $$OHR=\sum W_{ROGi}\times K_{OH+ROGi}$$

(3) $$E_{OH,j}=E_{j}\times OHR$$

(4) $$P_{i}=\frac{E_{OH,j}}{\sum_{k=1}^{n}E_{OH,k}}\times 100\%\;\;$$

where $W_{ROGi}$ is the molecular number concentration of the *i*-th ROG species (molecules·cm^−3^); $K_{OH+ROGi}$ is the reaction rate constant of the *i*-th ROG species with OH radicals (cm^3^·molecule^−1^·s^−1^); $E_{OH,j}$ represents the OH-weighted emission rate of industry *j* (g·s^−1^); $P_{i}$ is the proportion of OH-weighted emissions from industry *j* (%); and $\sum_{k=1}^{n}E_{OH,k}$ is the sum of OH-weighted emissions from all industries in the region, representing the OH consumption capacity of regional ROG species (g·s^−1^).

To further construct an OHR-weighted regional composite source profile, species-level weights were assigned based on both mass emission and OH reaction rate constants so that species with high emissions and high reactivity received greater weight in the integrated profile. The OH reaction rate constants used in this study were compiled from authoritative kinetic databases, including the NIST Chemical Kinetics Database, MCM v3.3.1, and IUPAC-recommended values. It should be noted that this OHR metric is emission-based and not directly correlated with ambient O_3_ formation.

## 3. Results and Discussion

### 3.1. Overall Compositional Characteristics of VOC Emissions from Typical Industries

A total of 123 VOC species were identified across five representative industrial sectors in the Chengdu–Chongqing region: pharmaceutical manufacturing, industrial coating, the chemical industry, food manufacturing, and the textile industry. These compounds were classified into seven chemical groups: alkanes, alkenes, alkynes, aromatics, halocarbons, OVOCs, and others. The absolute total VOC concentrations for each sampling point are provided in Table A1. Overall, the measured emissions were dominated by a few major species. Specifically, 68 species contributed less than 2% across all processes, whereas the remaining 55 dominant species accounted for 83.93–99.03% of the total identified VOCs, indicating that industrial VOC emissions in the region exhibit a pronounced skew toward a few key compounds.

**Figure 2:**
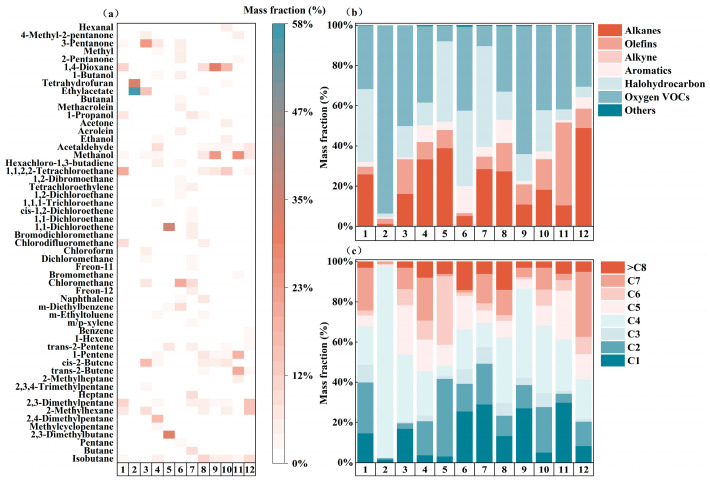
The number of carbon emissions from VOC in the five industries by process, the composition of the species and the dominant species. (**a**) Dominant species; (**b**) Chemical groups; (**c**) Carbon number distribution. Note: 1 PM-1: API production workshop; 2 PM-1: Theanine production workshop; 3 PM-2: Reactor workshop; 4 PM-3: Boiler exhaust outlet; 5 IP-1: RTO exhaust outlet; 6 IP-1: Activated carbon export; 7 IP-2: Powder coating workshop; 8 CM-1: Alkali washing import; 9 CM-1: Alkali washing outlet; 10 CM-2: Aggregation; 11 FM-1: Deep frying; 12 TX-1: Full-process production workshop.

Clear sector-specific compositional patterns were observed (Figure 2). Pharmaceutical manufacturing was characterized by a predominance of OVOCs, accounting for 31.61%, 38.01%, 93.66%, and 50.12% of total VOC across different workshops or outlets. In particular, ethyl acetate and tetrahydrofuran together accounted for more than 90% of the total VOC in the theanine production workshop, underscoring the strong influence of solvent use on source composition. Industrial coating, by contrast, was dominated by low-carbon compounds (C1–C4), with halocarbons consistently representing the largest chemical class (37.39–50.04%). Among them, 1,1-dichloroethylene accounted for 36.70% at the RTO outlet, while chloromethane contributed 20.43% and 9.68% at the activated carbon outlet and spray-coating workshop, respectively. These results suggest continued use of chlorinated solvents or related chlorinated materials in local coating processes.

The chemical industry displayed pronounced process dependence. Emissions from polymerization units were dominated by OVOCs (42.15%), while low-carbon species (C1–C4) accounted for 68.43% of the total VOCs. Methanol (16.04 ± 7.27%) and cis-2-butene (13.00 ± 4.71%) were the most abundant compounds, reflecting the combined influence of raw materials, intermediates, and process solvents. The food manufacturing and textile industries both exhibited mixed emissions, although with clearly different dominant species. Food manufacturing was enriched in alkenes and OVOCs, represented by trans-2-butene (20.59 ± 0.50%) and methanol (26.05 ± 0.26%), consistent with thermal decomposition and the release of oxygenated intermediates during frying or heating. Textile emissions, in contrast, were dominated by alkanes, with 2-methylhexane contributing 14.57 ± 3.31%, likely reflecting volatilization from cleaning agents, auxiliaries, or solvents, potentially compounded by inefficient local exhaust capture. Taken together, these results indicate that industrial VOC profiles in the Chengdu–Chongqing region are jointly shaped by process pathways, material usage, and emission collection and treatment conditions.

### 3.2. Inter-Industry Differences and Regional Heterogeneity in VOC Source Profiles

**Figure 3:**
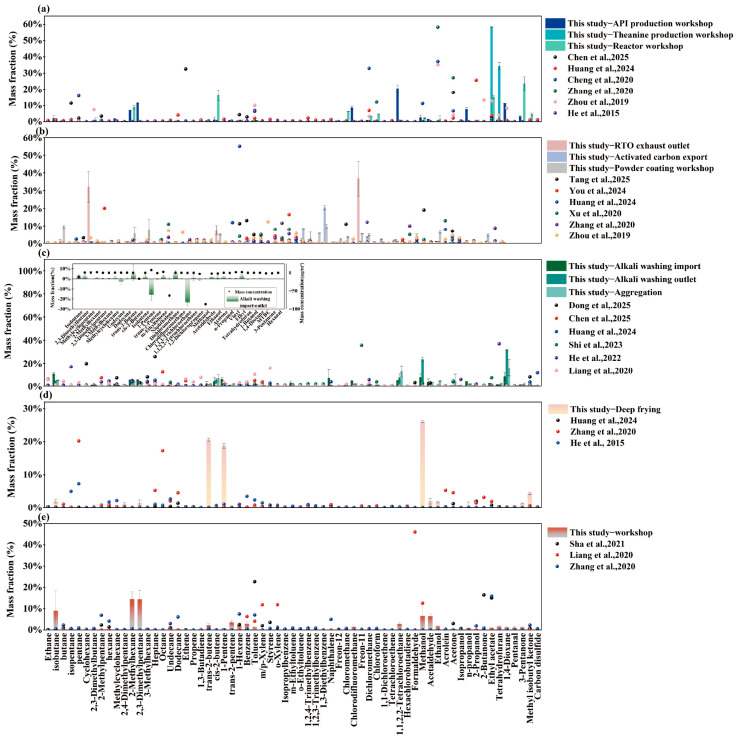
Research and Comparison of Dominant VOC Emission Species in Five Industries in Deyang City. (**a**) PM; (**b**) IP; (**c**) CM; (**d**) FM; (**e**) TX. [10,22,25,31,32,33,34,35,36,37,38,39,40,41].

To systematically demonstrate regional heterogeneity, the mass fractions of dominant chemical classes and top-ranking individual VOC species identified in our Deyang measurements were compared with source profiles from other Chinese regions reported in the recent literature (Figure 3). This cross-regional comparison revealed substantial spatial variability in source composition, although the extent of this heterogeneity varied distinctly across sectors. Pharmaceutical manufacturing and industrial coating showed relatively stable dominant chemical classes across regions, whereas the chemical industry, food manufacturing, and textile industry exhibited much stronger geographical variability.

In pharmaceutical manufacturing, OVOCs were consistently the dominant class, but the dominant individual compounds varied markedly among regions. The Deyang samples were characterized by 1,1,2,2-tetrachloroethane, ethyl acetate, and 3-pentanone, whereas previous studies have reported acetone-dominated profiles in Shanxi [31] and coastal areas [32]; ethanol-dominated profiles in Zhengzhou [33], the Yangtze River Delta [34], and Chengdu [35]. and elevated 2-propanol levels in Guiyang [22]. This pattern suggests that pharmaceutical emissions are relatively stable at the chemical-class level but highly variable at the species level, reflecting differences in product type, reaction pathway, and solvent system.

Industrial coating showed stronger cross-regional consistency, with halocarbons or other solvent-related compounds generally dominating most profiles. However, the Deyang samples were distinguished by chlorinated small molecules and branched alkanes, differing from profiles reported for Hangzhou [36], Zhengzhou [33], and northeastern China [10], where dichloromethane, tetrachloroethylene, n-hexane, and 1,2,4-trimethylbenzene were more prominent. This finding suggests that chlorinated solvents remain important in some heavy-manufacturing coating applications in the Chengdu–Chongqing region [35,37].

Among all sectors, the chemical industry showed the greatest heterogeneity, which is inherently linked to localized feedstocks and product structures. Cross-regional comparisons [22,25,31,38,39,40], showed that Deyang chemical plants were characterized by OVOCs such as methanol and reactive alkenes such as cis-2-butene, whereas ketones, tetrahydrofuran, aromatics, or alkanes dominated in other regions depending on local feedstocks, production routes, and product structures. Furthermore, our local measurements revealed how this heterogeneity is amplified by facility-specific treatments: the inlet of the alkali scrubbing unit contained a relatively diverse VOC mixture, whereas the outlet was more strongly enriched in methanol and naphthalene-likely due to the low removal efficiency of aqueous alkaline solutions for hydrophobic aromatics (naphthalene) and highly volatile alcohols (methanol)-with the contribution of the top five species increasing from 39.28% to 72.24%. This result indicates that end-of-pipe treatment may substantially reshape the composition of emitted VOC due to species-dependent removal efficiencies and chemical reactions within the control units (e.g., thermal oxidation in RTOs and photodegradation in UV photolysis systems, which actively destroy VOCs but may yield oxygenated intermediates), rather than simply reducing total VOC concentration. These findings underscore the limited representativeness of a generic “chemical industry” profile for regional emission characterization.

The food manufacturing and textile industries also showed substantial variability, although their differences appeared to be more closely linked to process conditions and plant management. In food manufacturing, dominant compounds varied across regions [22,32,33], likely due to differences in oil type, heating temperature, additives, and production conditions. In textile plants, the present study observed an alkane- and OVOC-rich profile with relatively low aromatic content, in contrast to the aromatic-rich emissions reported in previous studies [33,40,41]. Field observations suggest that this discrepancy may partly reflect differences in local collection efficiency and the volatilization of cleaning agents or auxiliaries. Overall, these results highlight the need for region-specific, process-resolved source profiles, especially in sectors with strong compositional variability.

### 3.3. Regional Composite Profile and OH Reactivity Characteristics

**Figure 4:**
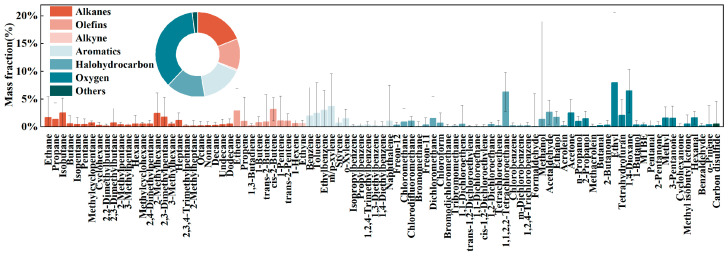
Emission-weighted regional aggregated source profile for VOC in the Chengdu–Chongqing region.

To characterize industrial VOC emissions at the regional scale, a composite source profile was constructed for 17 major industrial sectors using an emission-weighted integration approach (Equation (1); Figure 4). Total industrial VOC emissions were estimated at 1949.99 tons/year, and 93 dominant species, each contributing at least 1%, together accounted for 94.35% of the total. OVOCs were the most abundant chemical class (35.67%), followed by alkanes (19.01%) and aromatics (15.99%), indicating that industrial VOC emissions in the Chengdu–Chongqing region are shaped more strongly by oxygenated solvents and intermediates than by aromatics alone.

This regional composition exhibited a clear sector–species correspondence. OVOCs were contributed primarily by the chemical industry, printing, and pharmaceutical manufacturing; alkanes were associated mainly with the chemical industry and non-metallic mineral products; and aromatics were contributed mainly by printing and non-metallic mineral products. At the species level, the four most abundant compounds were ethyl acetate, 1,4-dioxane, 1,1,2,2-tetrachloroethane, and m/p-xylene. Ethyl acetate showed a clear cross-sector emission pattern, with the majority emitted from beverage/tea manufacturing, pharmaceutical manufacturing, and printing, reflecting its widespread use as a general solvent. In contrast, 1,4-dioxane and 1,1,2,2-tetrachloroethane were highly concentrated in the chemical industry, whereas m/p-xylene was strongly associated with printing. These results suggest that regional VOC control strategies should move beyond bulk mass reduction and instead distinguish between cross-sector common solvents and sector-specific signature species.

**Figure 5:**
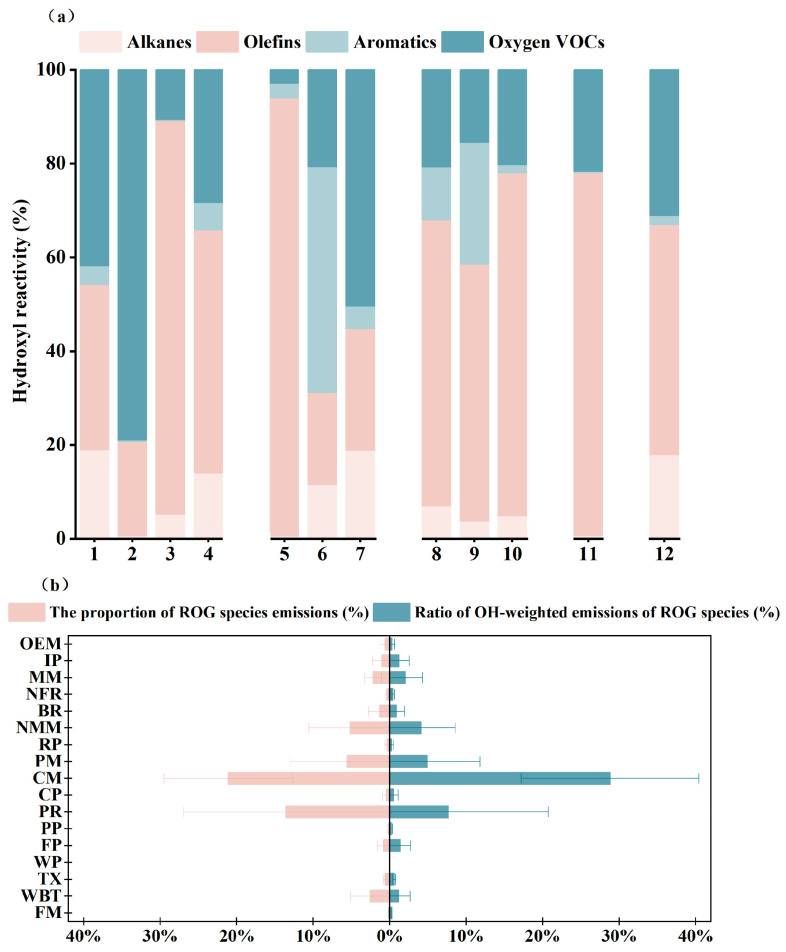
Analysis of Emissions and Reactivity of Industrial VOCs. (**a**) Composition of OH–reactive species from investigated processes; (**b**) Contribution of regional industrial sectors to mass emissions and OH–weighted emissions.

To further evaluate atmospheric implications, OHR was calculated for the measured industrial sources (Equation (2); Figure 5). Alkenes were the dominant contributors to OHR, accounting for 19.67–93.40% across processes, even though they were not always the most abundant compounds by mass. This pattern was particularly evident in the chemical industry and industrial coatings, where reactive alkenes contributed disproportionately to atmospheric oxidation potential. A clear decoupling between emission mass and chemical activity was also observed, a phenomenon increasingly recognized in recent atmospheric studies evaluating VOC reactivity [42]. For example, alkanes accounted for more than 40% of VOC mass in textile emissions but contributed less than 20% to OHR, whereas some lower-abundance alkenes made much larger contributions to OH loss. This mismatch highlights a central limitation of mass-based prioritization: species with the highest emissions are not necessarily those with the greatest atmospheric relevance.

A similar pattern emerged at the sectoral scale. The chemical industry contributed 21.10 ± 8.43% of reactive organic gas emissions but 28.82 ± 11.61% of OH-weighted emissions, indicating that it is both a major mass source and the leading driver of regional oxidation potential. In contrast, printing accounted for 13.55 ± 13.42% of reactive organic gas emissions but only 7.66 ± 13.08% of OH-weighted emissions, suggesting that its relative importance is overstated under a mass-based framework. Collectively, these results indicate that VOC management in the Chengdu–Chongqing region should shift from mass-oriented control to reactivity-oriented control [43], with priority given to high–OHR sectors and process units, particularly reactive alkene-rich operations in the chemical industry and industrial coating.

## 4. Conclusions

This study established source profiles for five representative industrial sectors in the Chengdu–Chongqing region and further developed a regional composite profile and OH-reactivity-based evaluation framework. The results revealed strong sector- and process-specific differences in VOC composition. Pharmaceutical manufacturing was generally dominated by OVOCs; industrial coating by low-carbon halocarbons; the chemical industry by methanol and other reactive low-carbon compounds; food manufacturing by alkenes and OVOCs; and the textile industry by light alkanes. Cross-regional comparisons further showed that pharmaceutical manufacturing and industrial coating were relatively stable at the chemical-class level, whereas the chemical industry, food manufacturing, and the textile industry exhibited much stronger regional heterogeneity.

At the regional scale, industrial VOC emissions were dominated by OVOCs (35.67%), followed by alkanes (19.01%) and aromatics (15.99%). The most abundant species were ethyl acetate, 1,4-dioxane, 1,1,2,2-tetrachloroethane, and m/p-xylene, representing both cross-sector general solvents and sector-specific signature compounds. These results indicate that regional VOC control should move beyond bulk emission reduction and instead focus on sector–species linkages.

From an atmospheric chemistry perspective, OHR was largely controlled by alkenes, and a pronounced mismatch was observed between emission mass and chemical activity. The chemical industry was not only a major VOC source by mass but also the leading contributor to OH-weighted emissions, whereas printing showed the opposite pattern. Effective VOC control in the Chengdu–Chongqing region, therefore, requires a shift from concentration-oriented to reactivity-oriented management, prioritizing high-activity sectors and reactive process units to enhance the ozone-relevant benefits of VOC control, noting that the OHR-weighted metric is intended for source prioritization rather than direct O_3_ prediction.

It should be noted that uncertainties remain in estimating regional emission contributions. Key variables in this study include VOC mass fractions, emission rates, OHR and process-level parameters such as exhaust volume and treatment technology. Our approach combines limited field measurements and literature profiles with a bottom-up inventory. While this improves regional representativeness, extrapolating from a subset of sampled enterprises may not fully reflect intra-sector variability. Future work should expand sampling across more diverse operating conditions and sub-sectors to reduce these uncertainties.

## Figures and Tables

**Table 1:** Sub-industry types and enterprise sampling information.

Enterprise Code	Sampling Point	Number of Samples	Organic Waste Gas Collection Device	Organic Waste Gas Treatment Process
PM-1	API production workshop	2	enclosed pipeline collection	UV photolysis + water spray absorption + activated carbon adsorption
PM-2	Theanine production workshop	1	enclosed pipeline collection	alkali scrubbing + dry filtration + UV photolysis + activated carbon adsorption
	Reactor workshop	1	enclosed pipeline collection	alkali scrubbing + dry filtration + UV photolysis + activated carbon adsorption
PM-3	Boiler exhaust outlet	2	enclosed pipeline collection	activated carbon adsorption
IP-1	RTO exhaust outlet	1	enclosed pipeline collection	dry filtration + zeolite rotor + RTO
	Activated carbon export	1	enclosed pipeline collection	activated carbon adsorption
IP-2	Powder coating workshop	1	enclosed spray painting booth	filtering cotton + UV photolysis
CM-1	Alkali washing import	2	fume hood	alkali scrubbing + acid washing + activated carbon adsorption
	Alkali washing outlet	2	fume hood	alkali scrubbing + acid washing + activated carbon adsorption
CM-2	Aggregation	2	enclosed pipeline collection	condensation + alkali scrubbing + activated carbon adsorption
FM-1	Deep frying	2	fume hood	high-efficiency oil fume purifier + UV photolysis
TX-1	Printing and Dyeing Production Workshop	2	enclosed pipeline collection	spray absorption

Note: Enterprise Code indicates the specific facility sampled. The letters represent the sector abbreviation (e.g., PM for Pharmaceutical Manufacturing), and the number represents the specific plant sampled within that sector.

**Table 2:** Sources of VOC species profiles in the Chengdu–Chongqing region.

No.	Industry Name	Industry Code	Emissions (Tons/Year)	Profile Source	Notes/References
1	FM	C13–14	4.13	Measured—Frying Process	This study
2	WBT	C15	91.17	Literature Review	Huang et al., 2024 [22]
3	TX	C17	17.62	Measured—Printing and Dyeing Production Workshop	This study
4	WP	C20	0.18	Literature Review	Du et al., 2024 [23]
5	FP	C21	36.88	Literature Review	Huang et al., 2024 [22]
6	PP	C22	26.64	Literature Review	EPA [24]
7	PR	C23	414.88	Literature Review	Shi et al., 2023 [25]
8	CP	C25	15.32	Literature Review	Zhu et al., 2024 [11]
9	CM	C26	735.91	Measured—Polymerization Process	This study
10	PM	C27	185.65	Measured—Production Workshop	This study
11	RP	C29	10.54	Literature Review	Wu et al., 2017 [26]
12	NMM	C30	195.91	Literature Review	He et al., 2017 [27]
13	BR	C31	60.26	Literature Review	He et al., 2017 [27]
14	NFR	C32	12.55	Literature Review	Han et al., 2020 [28]
15	MM	C33	70.01	Literature Review	Mo et al., 2015 [29]
16	IP	C34–36	49.76	Measured—Spraying Workshop	This study
17	OEM	C37–39	22.58	Literature Review	Wu et al., 2017 [26]

Note: No. (1–17) represents the sequential identifier for the broad industrial sectors evaluated at the regional level. “This study” refers to the measured values from five industries (FM, TX, CM, PM, IP). “Industry codes” follow GB/T 4754-2017 [30] (C13–C39); based on Deyang’s local industrial structure, C13–14 are merged into FM, C34–36 into IP, and C37–39 into OEM. “Measured” denotes our own experimental data, while “Literature Review” denotes data from previous studies.

## Data Availability

The original contributions presented in this study are included in the article. Further inquiries can be directed to the corresponding author.

## Abbreviations

The following abbreviations are used in this manuscript:

VOCs	volatile organic compounds
OVOCs	oxygenated volatile organic compounds
OH	hydroxyl radical
PM_2.5_	fine particulate matter
O_3_	ozone
FM	Food Manufacturing Industry
WBT	Manufacture of Wine, Beverages and Refined Tea Industry
TX	Textile Industry
WP	Wood Processing Industry
FP	Furniture Manufacturing Industry
PP	Paper and Paper Products Manufacturing Industry
PR	Printing and Reproduction of Recording Media Industry
CP	Crude Oil Processing and Petroleum Products Manufacturing Industry
CM	Chemical Industry
PM	Pharmaceutical Manufacturing Industry
RP	Rubber and Plastic Products Manufacturing Industry
NMM	Manufacture of Non-metallic Mineral Products Industry
BR	Smelting and Pressing of Ferrous Metals Industry
NFR	Smelting and Rolling of Non-ferrous Metals
MM	Metal Products Manufacturing Industry
IP	Industrial Coating Industry
OEM	Other Equipment Manufacturing Industry
API	Active Pharmaceutical Ingredient

## Appendix A

**Table A1:** Absolute total VOC concentrations at each sampling point.

Enterprise Code	Sampling Point	Total VOC Concentration (mg/m^3^)	SD
PM-1	API production workshop	0.08	0.00
		0.06	0.01
PM-2	Theanine production workshop	7.85	1.89
	Reactor workshop	1.50	0.18
PM-3	Boiler exhaust outlet	0.24	0.04
		0.21	0.02
IP-1	RTO exhaust outlet	1.30	0.28
	Activated carbon export	1.32	0.08
IP-2	Powder coating workshop	0.21	0.00
CM-1	Alkali washing import	0.11	0.03
		0.15	0.01
	Alkali washing outlet	0.33	0.04
		0.29	0.01
CM-2	Aggregation	0.23	0.04
		0.32	0.01
FM-1	Deep frying	1.50	0.09
		1.63	0.26
TX-1	Printing and Dyeing Production Workshop	0.53	0.10
		0.62	0.18
